# Influence of caffeine on the maximal isometric and concentric force produced by skinned fibers

**DOI:** 10.1038/s41598-022-12222-4

**Published:** 2022-05-13

**Authors:** Atsuki Fukutani, Shiho Kunimatsu, Tadao Isaka

**Affiliations:** 1 Faculty of Sport and Health Science, Ritsumeikan University, 1-1-1 Noji-higashi, Kusatsu, Shiga 525-8577 Japan; 2 College of Sport and Health Science, Ritsumeikan University, 1-1-1 Noji-higashi, Kusatsu, Shiga 525-8577 Japan

**Keywords:** Bone quality and biomechanics, Medical research

## Abstract

Caffeine is one of the most famous and widely used ergogenic drugs, especially by athletes to improve sports performance. Caffeine is known to enhance muscle contraction by facilitating Ca^2+^ release from the sarcoplasmic reticulum. While the effect of caffeine on the cross-bridge dynamics has also investigated, the results is controversial. Therefore, the purpose of this study was to examine the influence of caffeine on cross-bridge dynamics using skinned fiber preparations from rabbit soleus (N = 19 in total). We performed isometric contractions at an average sarcomere length of 2.4 μm; thereafter, skinned fibers were shortened by 20% of the fiber length at a velocity of 0.1 mm/s (slow shortening) or 0.5 mm/s (fast shortening). The contractions were performed under both normal and caffeine-containing activating solution conditions to compare the isometric, slow concentric, and fast concentric forces between conditions. The isometric force did not differ between normal and caffeine-containing activating solution conditions. Similarly, the concentric forces obtained during the slow and fast shortening trials did not differ between conditions. We also measured the stiffness and the rate of force redevelopment (kTR) during the isometric contraction phase and found that these values were not different between normal and caffeine conditions. Based on these results, we conclude that the influence of caffeine on cross-bridge dynamics is negligible, and the ergogenic effect of caffeine, from the view of muscle contractility, is by facilitating Ca^2+^ release, as suggested in previous studies, and not by modulating the cross-bridge dynamics.

## Introduction

Caffeine is commonly consumed by many people because of its presence in coffee, tea, and cocoa^1^. Caffeine has several effects on humans, including reducing drowsiness; similarly, caffeine enhances neuronal activity^2^. In fact, due to this excitation effect, the World Anti-Doping Agency identified caffeine as a doping substance. However, this rule was recently withdrawn^3^; as a result, various kinds of caffeine-containing “energy drinks” or supplements are widely used^4^.

Because of its ergogenic effect, caffeine is used especially among athletes. In addition to the aforementioned psychiatric effects, caffeine is known to enhance muscle contraction. Black et al.^5^ showed that a 5 mg/kg dose of caffeine increased the maximal isometric torque in knee extensors in humans. In addition, Tarnopolsky et al.^6^ reported that in a fatiguing protocol, a 6 mg/kg dose of caffeine ingestion alleviated a reduction in electrically stimulated tetanic torque using low-frequency stimulation (20 Hz). At present, the widely accepted mechanism for the enhancement by caffeine is the facilitation of Ca^2+^ release from the sarcoplasmic reticulum^7,8^. An increased Ca^2+^ release from the sarcoplasmic reticulum leads to the release of tropomyosin, which prevents actin and myosin interactions; consequently, more cross-bridges can attach to the actin filaments.

Recently, Neyroud et al.^9^ used single intact fiber preparations and demonstrated that the isometric force increased with 1 mM caffeine perfusion and the increase was accompanied by an increase in intramuscular Ca^2+^ concentration. This result supports the well-known mechanism of caffeine activation, i.e., the facilitation of Ca^2+^ release from the sarcoplasmic reticulum. If this is a sole mechanism for enhancing muscle contractions, we could not expect the increase in the maximal force/torque because the Ca^2+^ concentration is saturated in the high intensity contractions. However, if caffeine also enhances cross-bridge dynamics directly, independent of Ca^2+^ release, there is a possibility that caffeine can enhance even the maximal intensity contractions. Although the effect of caffeine on Ca^2+^ release has been widely examined and well-established, the effect of caffeine on cross-bridge dynamics has remained controversial. To examine this point, chemically skinned fiber preparations are ideal because the sarcoplasmic reticulum does not work in these fibers, and the Ca^2+^ concentration can be easily controlled, artificially. Using these fiber preparations, the effect of caffeine on the sarcoplasmic reticulum is excluded, and consequently, the effect of caffeine on cross-bridge dynamics could be examined directly. Therefore, the purpose of this study was to examine whether caffeine affects cross-bridge dynamics using skinned soleus muscles from rabbits.

## Methods

### Muscle samples and experimental setups

We purchased isolated rabbit muscle tissues from SHIMIZU Laboratory Supplies. They harvested these muscle tissues from a New Zealand white rabbit. These processes were conducted according to the Guidelines for Proper Conduct of Animal Experiments (since June 1, 2006), and approved by Japanese Society for Laboratory Animal Resources (17-026). We adopted the soleus muscles, which are mainly composed of slow twitch fibers^10^, to minimize the influence of fatigue and/or damage. Strips of the soleus muscles were harvested and tied to wooden sticks to preserve the in situ sarcomere length. The strips were then placed in a 50% rigor:50% glycerol solution with protease inhibitors (cOmplete™, Roche Sigma-Aldrich, US) to chemically disrupt the muscle membrane. Subsequently, the strips were stored in a freezer at −20 °C for more than 2 weeks. On the day of the experiments, a single fiber or small bundles (at most, three fibers) of the soleus muscle were isolated using fine forceps under a dissecting microscope (SM-1TSW2-L6W-M, AmScope, US). The isolated fiber was transferred to a custom-made experimental chamber containing a relaxing solution with protease inhibitors (cOmplete™, Roche Sigma-Aldrich, US). One end of the fiber was attached to a force transducer (Experiment 1; UL-2GR, MinebeaMitsumi, Japan, Experiment 2; 403B, Aurora Scientific, Canada) mounted on a length controller (Experiment 1; DRSM42RG-04B2AZAK, Oriental Motor, Japan, Experiment 2; 315D, Aurora Scientific, Canada), and the other end was fixed to a rigid metal hook by using silk string. The sarcomere length was measured using a He–Ne laser-based diffraction system (HNLS008L-JP, THORLABS, Japan). Fiber or bundle length (1.3 ± 0.5 mm for experiment 1 and 1.9 ± 0.1 mm for experiment 2) and fiber or bundle diameter (82.0 ± 22.0 μm for experiment 1 and 75.8 ± 11.6 μm for experiment 2) were measured using a stereo microscope (SM-8TW2-144S, AmScope, US). Room temperature was controlled within a range of 23.0–24.3 °C for experiment 1 and 26.7–27.1 °C for experiment 2. The experimental solutions were used under these room temperature conditions.

### Experimental procedures and measurements

We conducted two experiments in this study. Skinned fibers of rabbit soleus muscle (N = 10 from one rabbit for experiment 1 and N = 9 from a different rabbit for experiment 2) were used in this study. The same fiber was used for both the normal and caffeine conditions.

**Figure 1:**
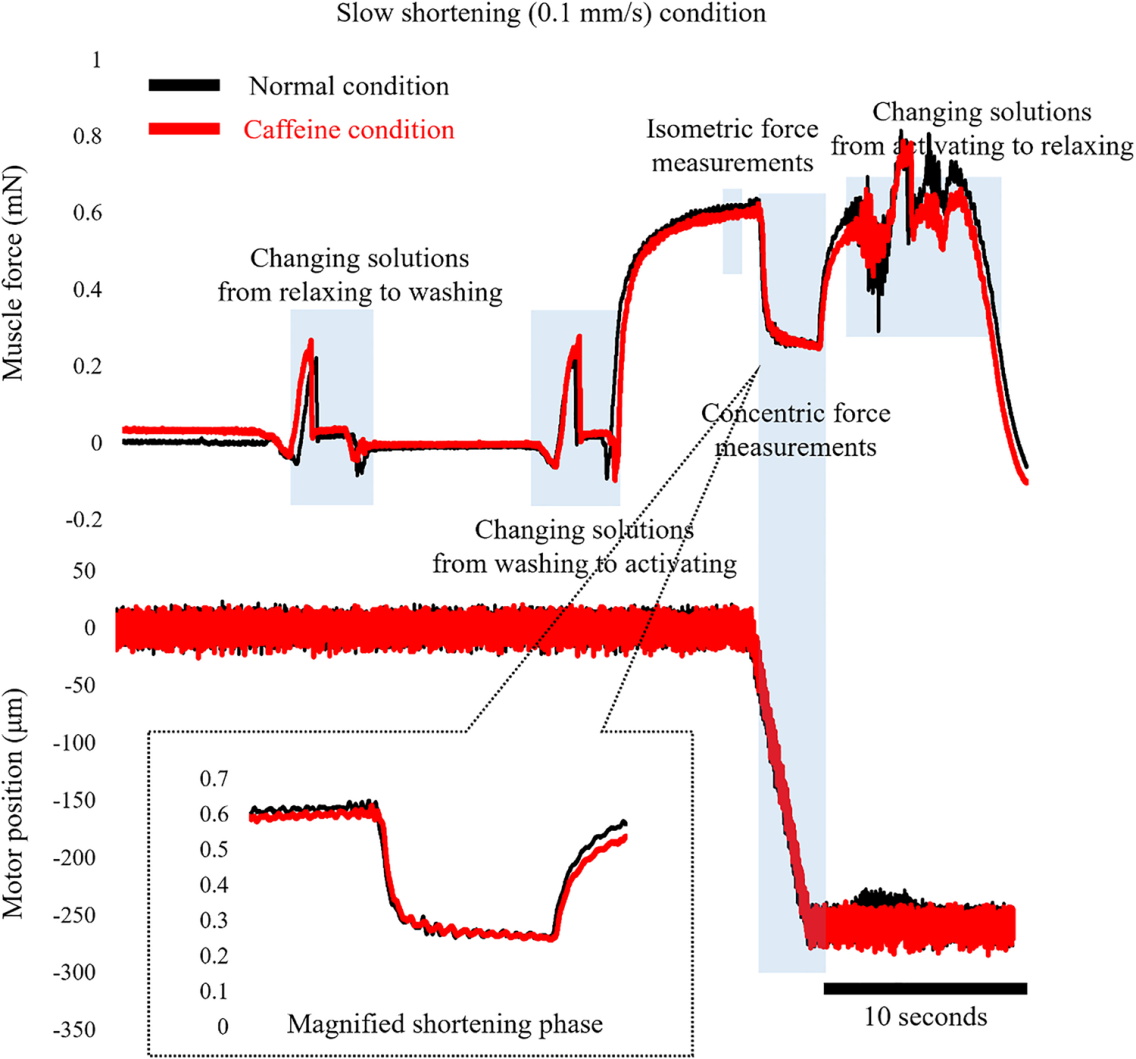
Force and length responses for the normal and caffeine conditions in the slow shortening trials. Black lines indicate the normal condition and red lines indicate the caffeine condition. The inset shows the magnified force responses during the shortening phase.

**Figure 2:**
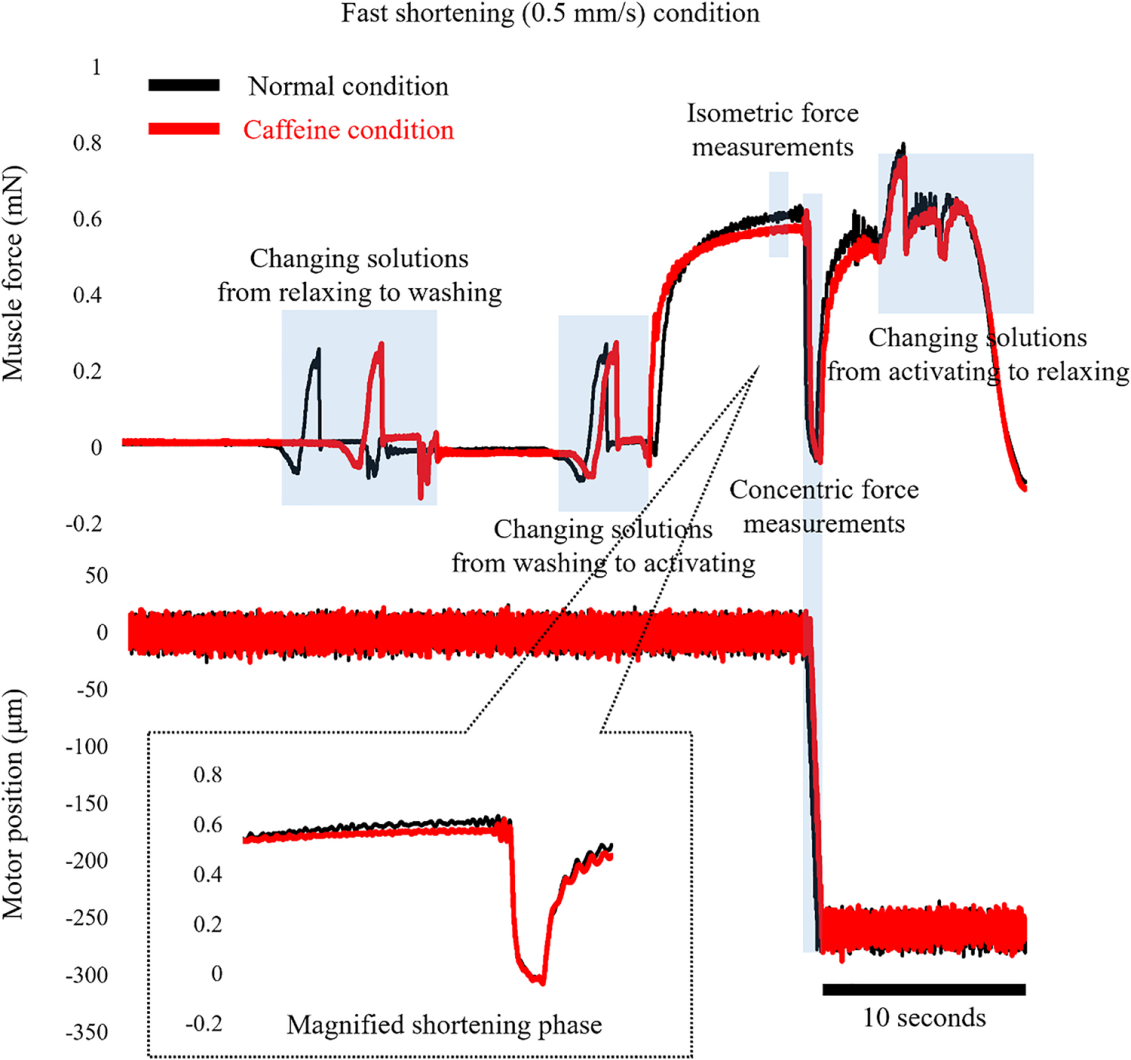
Force and length responses for the normal and caffeine conditions in the fast shortening trials. Black lines indicate the normal condition and red lines indicate the caffeine condition. The inset shows the magnified force responses during the shortening phase.

In experiment 1, normal activating solution (normal condition) and caffeine-containing activating solution (caffeine condition) conditions were adopted, and each of these conditions was composed of slow shortening and fast shortening trials (Figs. 1, 2). In the slow shortening trial, fibers were activated at an average sarcomere length of 2.4 μm, and once the isometric force became stable, fibers were actively shortened at a velocity of 0.1 mm/s. The shortening magnitude was 20% of the fiber length. In the fast shortening trial, the protocol was the same, except for the shortening velocity, which was 0.5 mm/s. Under normal conditions, fibers were activated using the normal activating solution, whereas fibers were activated using a caffeine-containing activating solution in the caffeine condition (described below in the “solutions” section). The order of the normal and caffeine conditions, and that of the slow and fast trials, was randomized. Each test was separated by at least two minutes. Force and length data were collected at 1000 Hz. The maximal isometric force was obtained immediately before initiating subsequent shortening. The concentric force was evaluated as the mean value of the force attained during the shortening phase (Fig. 1, 2). These values obtained in the slow and fast trials were compared between normal and caffeine conditions.

**Figure 3:**
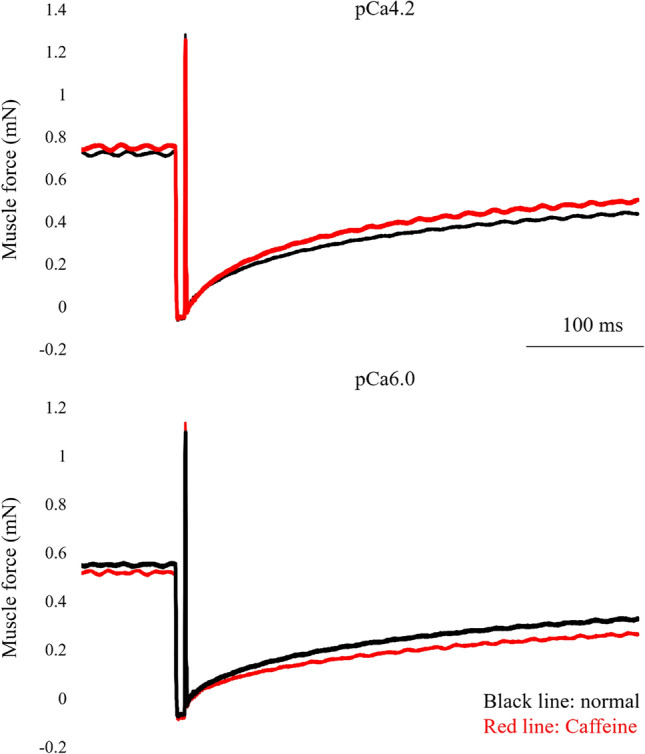
Force responses for the normal and caffeine conditions in the kTR protocol (force declines to zero by a quick shortening and redevelops by a subsequent quick restretch to the initial length). Black lines indicate the normal condition and red lines indicate the caffeine condition.

In experiment 2, in addition to the isometric force, stiffness and rate of force redevelopment (kTR) were obtained in the maximal Ca^2+^ concentration (pCa 4.2) and the submaximal Ca^2+^ concentration (pCa 6.0) states between normal and caffeine conditions to evaluate the cross-bridge behaviors. Fibers are activated at an average sarcomere length of 2.4 μm. Once the isometric force became stable, fibers were quickly stretched by 0.2% of the fiber length in 1 ms to measure the stiffness of the fibers. Then, fibers were quickly shortened by 10% of the fiber length to decrease the force to zero. After that, fibers were quickly stretched to the initial sarcomere length (2.4 μm) to measure the rate of force redevelopment (kTR) (Fig. 3). kTR was calculated as follows; log2/t_1/2_ where t_1/2_ is the time to the 50% of force redevelopment according to the previous study (Lee et al. 2010). This test was performed in the both normal and caffeine conditions and the same test was also performed in the submaximal intensity contractions (i.e., the normal activating solution with pCa 6.0 and the caffeine-containing activating solution with pCa 6.0).

### Solutions

The solution recipes, except for the caffeine-containing activating solution, were the same as that of Fukutani et al.^11,12^. Specifically, the relaxing solution contained 170 mM potassium propionate, 2.5 mM magnesium acetate, 20 mM MOPS, 5 mM K_2_EGTA, and 2.5 mM ATP at pH 7.0. The washing solution contained 185 mM potassium propionate, 2.5 mM magnesium acetate, 20 mM MOPS, and 2.5 mM ATP at pH 7.0. The normal activating solution contained 170 mM potassium propionate, 2.5 mM magnesium acetate, 10 mM MOPS, 2.5 mM ATP, and free Ca^2+^ buffered with EGTA (CaEGTA and K_2_EGTA mixed to obtain a pCa value of 4.2) at pH 7.0. The caffeine-containing activating solution was the same as that of the normal activating solution, except for 10 mM caffeine. For experiment 2, lower pCa solutions (the normal activating solution with pCa 6.0 and the caffeine-containing activating solution with pCa 6.0) were prepared by adjusting the concentration of CaEGTA and K_2_EGTA . One tablet of protease inhibitor (cOmplete™, Roche Sigma-Aldrich, US) was added to each 100 mL of relaxing solution.

### Statistical analysis

Descriptive data are presented as means ± standard deviation (SD). Muscle force was expressed as stress by dividing the cross-sectional area of fibers (assuming cylindrical shape). To examine the difference between normal and caffeine conditions, a paired t-test was used for the magnitude of isometric force and slow and fast concentric forces, stiffness, and kTR. For each t-test, the effect size was calculated as Cohen's d. The level of significance was set at P < 0.05. Statistical analyses were conducted using IBM SPSS Statistics version 27.

## Results

**Figure 4:**
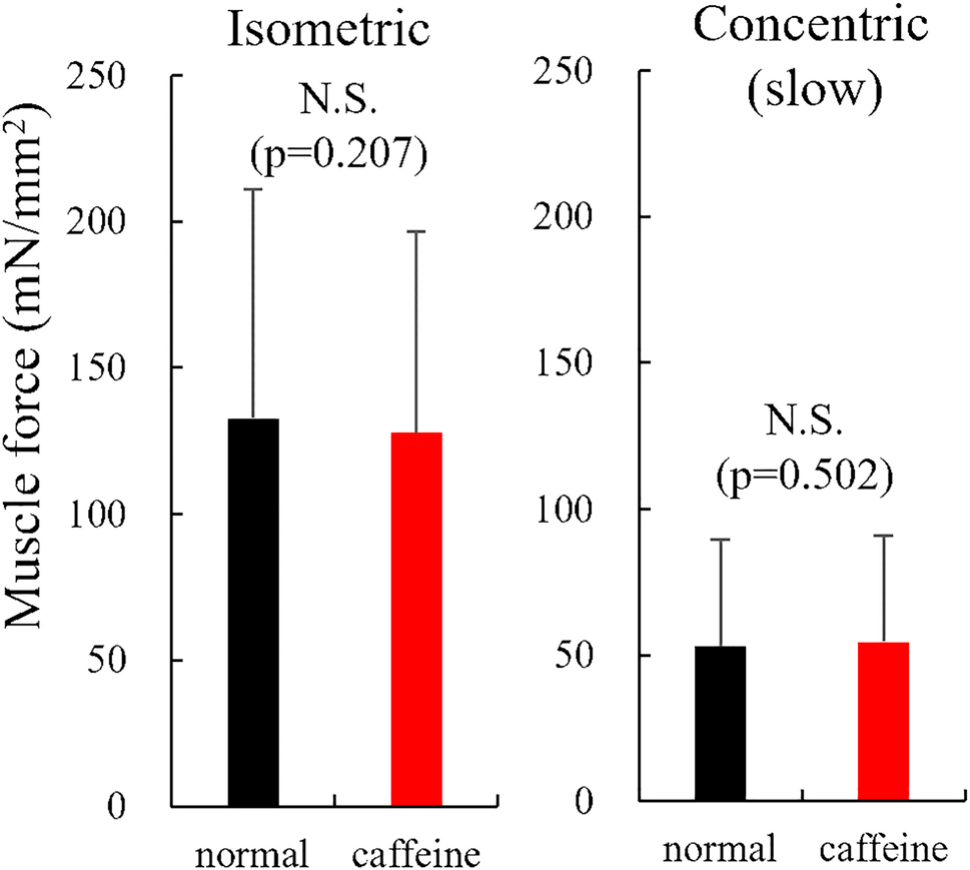
Muscle force obtained during the isometric and concentric phases in the slow shortening trials between normal and caffeine conditions (N = 10) (values are means ± SD).

**Figure 5:**
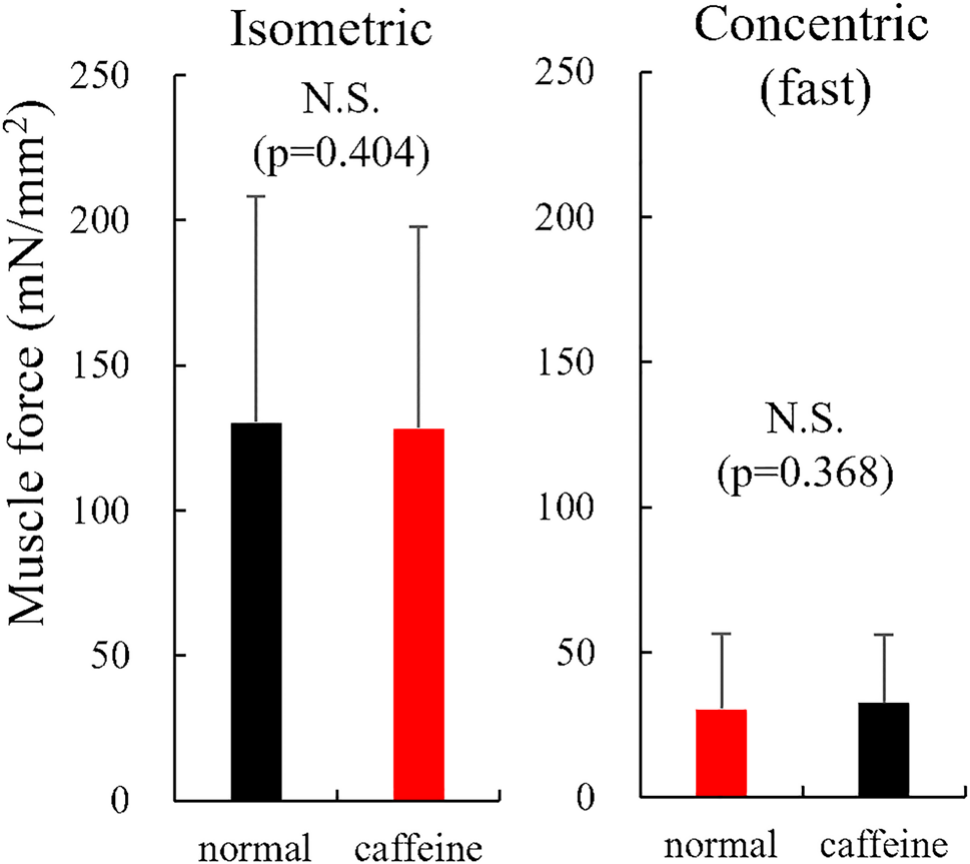
Muscle force obtained during the isometric and concentric phases in the fast shortening trials between normal and caffeine conditions (N = 10) (values are means ± SD).

For experiment 1, the isometric force was not significantly different (p = 0.207, effect size = 0.066) between normal (132.6 ± 78.5 mN/mm^2^) and caffeine (127.7 ± 68.8 mN/mm^2^) conditions in the slow shortening trial (Fig. 4). The slow concentric force was also not significantly different (p = 0.502, effect size = 0.040) between normal (53.1 ± 36.6 mN/mm^2^) and caffeine (54.5 ± 36.4 mN/mm^2^) conditions. Regarding the fast shortening trial, the isometric force was not significantly different (p = 0.694, effect size = 0.026) between normal (130.3 ± 78.0 mN/mm^2^) and caffeine (128.4 ± 69.3 mN/mm^2^) conditions (Fig. 5). Furthermore, the fast concentric force was not significantly different (p = 0.328, effect size = 0.089) between the normal (30.4 ± 26.1 mN/mm^2^) and caffeine (32.6 ± 23.4 mN/mm^2^) conditions.

**Figure 6:**
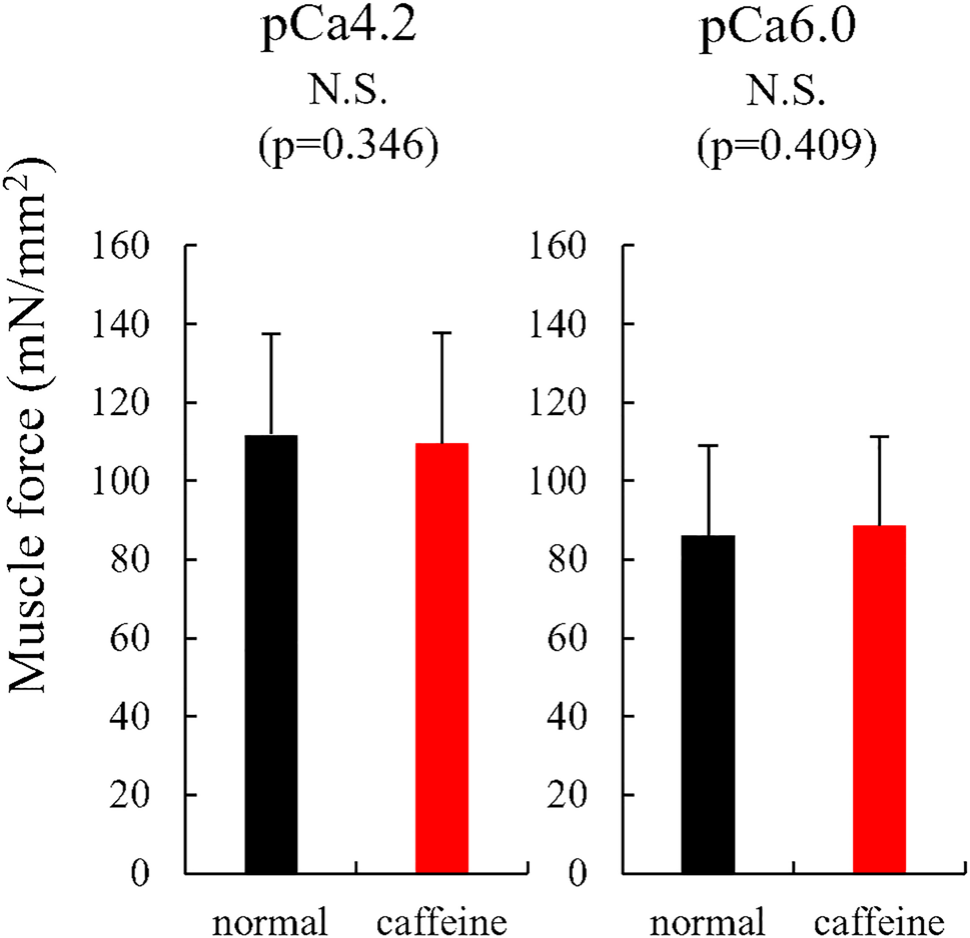
Muscle force obtained during the isometric phase (just before the stiffness test) for experiment 2 between normal and caffeine conditions (N = 9) (values are means ± SD).

**Figure 7:**
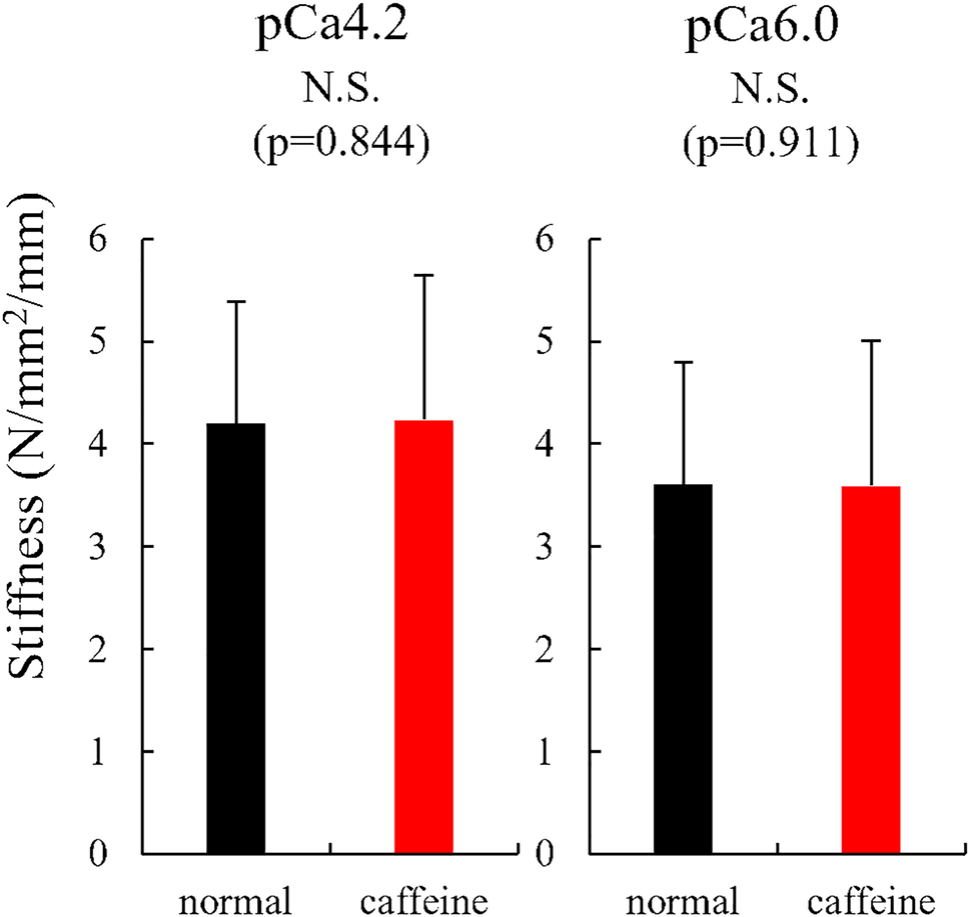
Stiffness obtained during the isometric phase for experiment 2 between normal and caffeine conditions (N = 9) (values are means ± SD).

**Figure 8:**
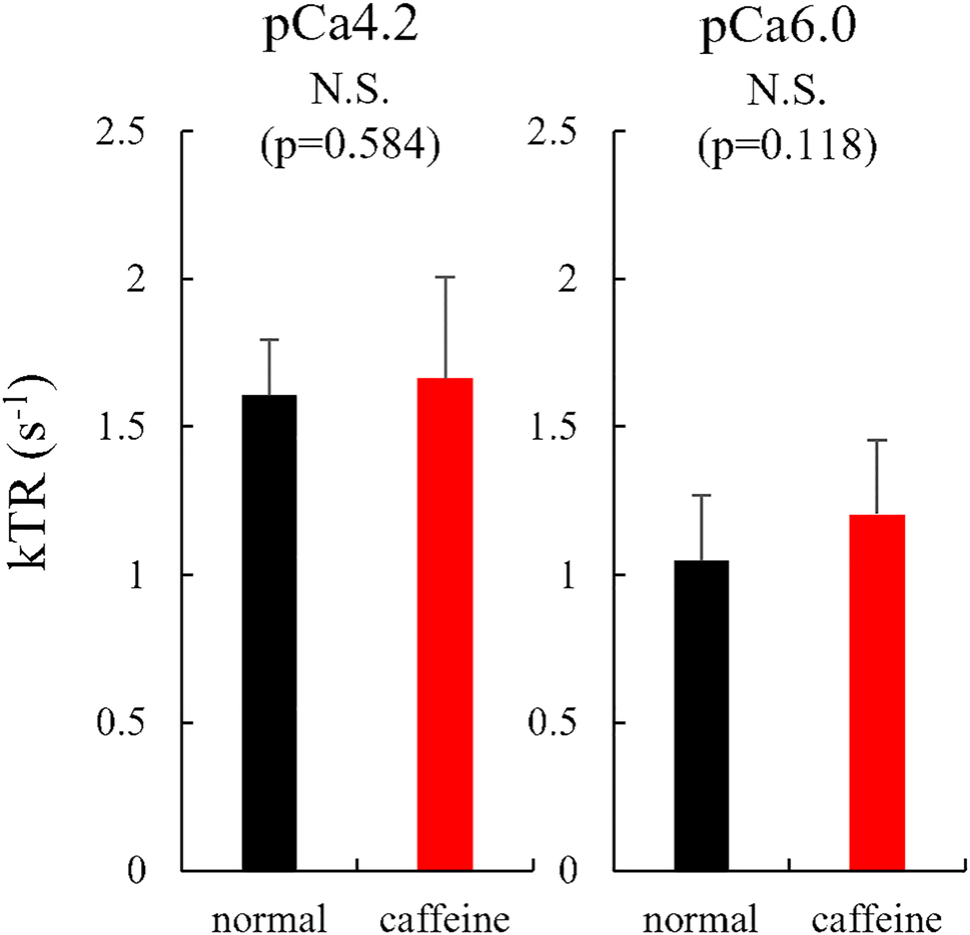
kTR obtained by a quick shortening and the subsequent quick restretch for experiment 2 between normal and caffeine conditions (N = 9) (values are means ± SD).

For experiment 2, the isometric force was not different between normal and caffeine conditions (111.7 ± 25.7 mN/mm^2^ for the normal condition and 109.5 ± 28.2 mN/mm^2^) (p = 0.346, effect size = 0.080) and the similar result was observed in the submaximal (pCa 6.0) condition (86.2 ± 22.7 mN/mm^2^ for the normal condition and 88.7 ± 22.7 mN/mm^2^ for the caffeine condition) (p = 0.409, effect size = 0.111) (Fig. 6). The stiffness attained during the isometric contraction was not different between normal and caffeine condition (4.2 ± 1.2 N/mm^2^/mm for the normal condition and 4.2 ± 1.4 N/mm^2^/mm for the caffeine condition) (p = 0.844, effect size = 0.026). The stiffness attained in the submaximal (pCa 6.0) condition was not different between normal and caffeine condition (3.6 ± 1.3 N/mm^2^/mm for the normal condition and 3.6 ± 1.3 N/mm^2^/mm for the caffeine condition) (p = 0.911, effect size = 0.009) (Fig. 7). The kTR was not different between normal and caffeine condition (1.61 ± 0.19 s^−1^ for the normal condition and 1.66 ± 0.34 s^−1^ for the caffeine condition) (p = 0.584, effect size = 0.203), and the kTR attained in the submaximal (pCa 6.0) condition was not different between normal and caffeine condition (1.05 ± 0.22 s^−1^ for the normal condition and 1.21 ± 0.25 s^−1^ for the caffeine condition) (p = 0.118, effect size = 0.631) (Fig. 8).

## Discussion

The purpose of this study was to examine whether caffeine affects cross-bridge dynamics using skinned fiber preparations. We found that adding 10 mM caffeine to the activating solution did not affect the maximal isometric force or the slow and fast maximal concentric forces. We also calculated the effect size for each comparison, and the values were very small (0.026–0.089). In addition, the stiffness as an index of the number of attached cross-bridge and kTR which depends on the rate constant of attachment and detachment of cross-bridge were not different between normal and caffeine conditions. These results strongly suggest that caffeine does not affect cross-bridge dynamics during maximal intensity contractions.

It is widely considered that caffeine enhances force-generating capability. Neyroud et al.^9^ found that using an intact single fiber, muscle force was increased by adding caffeine, and this increment was accompanied by an increase in intramuscular Ca^2+^ concentration. This result supports the current paradigm that the mechanism for the increase in force is the facilitation of Ca^2+^ release from the sarcoplasmic reticulum. If Ca^2+^ release is facilitated, muscle force should be enhanced because more cross-bridges can attach to actin filaments. Based on this mechanism, the effect of caffeine should be prominent when the Ca^2+^ concentration is low, and the effect should become small or negligible when the Ca^2+^ concentration is high enough to produce the maximal force. However, if caffeine also directly affects cross-bridge dynamics, it can also enhance high-intensity contractions via a mechanism that is independent of enhanced Ca^2+^ release. Based on this, we examined the effect of caffeine on cross-bridge dynamics using skinned fiber preparations, which eliminates the effect of caffeine on Ca^2+^ release; however, 10 mM caffeine did not alter the maximal isometric force or maximal concentric force. Therefore, the number of attached cross-bridges, force per attached cross bridges, and rate constant of attachment/detachment are not affected substantially by caffeine ingestion. We adopted a relatively high concentration of caffeine (10 mM), which is much higher than the physiological concentration (about 0.04–0.07 mM^13,14^). Thus, it would be safe to assume that physiological caffeine ingestion in humans does not enhance the force-generating capability from the viewpoint of cross-bridge dynamics at maximal intensity contractions.

As discussed above, it is likely that the effect of caffeine on muscle contractions would be limited for the relatively low intensity (low Ca^2+^ concentration) contractions. Based on the well-known characteristics of caffeine, that is, facilitating the Ca^2+^ release from the sarcoplasmic reticulum^7,8^, high-intensity (high Ca^2+^ concentration) contractions should not be enhanced by caffeine. Facilitating Ca^2+^ release is effective only when the Ca^2+^ concentration is not sufficient to induce the maximal force. It is well known that muscle force increases as the Ca^2+^ concentration increases, but this increase in force is saturated at a given Ca^2+^ concentration^15^. In addition, we found that caffeine did not affect the cross-bridge dynamics in both isometric and concentric contractions. Therefore, the maximal force at which the Ca^2+^ concentration should be saturated should not be enhanced by caffeine ingestion. If this is correct, some contradictions among previous studies regarding the effect of caffeine on muscle contractions can be explained. Although several studies reported the positive effect of caffeine on muscle contractions^6,9^, contradicting results reporting no enhancement also exist. For example, Trevino et al.^16^ reported that the maximal isometric torque in elbow flexors was not enhanced by 0, 5, or 10 mg of caffeine/kg of body mass in humans. In addition, Williams et al.^17^ reported that 7 mg of caffeine /kg of body mass did not enhance the maximal isometric hand grip force. These studies reporting no enhancement by caffeine ingestion adopted the maximal intensity contraction. In this case, the Ca^2+^ concentration must have been saturated. Therefore, it is difficult to expect an increased force due to caffeine, at least from the perspective of muscle contractility.

Our results are partly in line with a previous study reporting that caffeine (5–40 mM) did not increase the maximal isometric force obtained from cardiac and skeletal skinned fibers^18^. In our study, the maximal concentric force was also examined in addition to the maximal isometric force, and found the similar result that the maximal concentric forces attained in both slow and fast shortening were not increased by adding caffeine. While our study adopted the 10 mM concentration of caffeine, Wendt and Stephenson^18^ adopted several caffeine concentrations; interestingly, they reported that when the caffeine concentration was 40 mM, the maximal isometric force was decreased. Similarly, Powers and Solaro^19^ reported that although 20 mM caffeine increased isometric submaximal forces, the maximal isometric forces were depressed in skinned fiber preparations. Based on these previous studies and our current results, it is difficult to expect force enhancement in the maximal intensity isometric or concentric contractions, even if the caffeine concentration is increased.

Because the isometric force and concentric force were not changed by caffeine, it is likely that caffeine would not affect the cross-bridge dynamics. To strengthen this concept, we also measured stiffness and kTR in the pCa 4.2 and pCa 6.0 conditions (experiment 2). As with experiment 1, the isometric force was not different between normal and caffeine conditions. Similarly, stiffness and kTR were not different between conditions. Stiffness is known to represent the number of attached cross-bridge^20^ and kTR depends on the rate of attachment and detachment of cross-bridge^21^. These results indicate that 10mM caffeine does not affect the cross-bridge dynamics. However, other indexes which are related to cross-bridge dynamics were not measured in this study. To further strengthen our conclusion, these parameters should be measured in future studies.

In conclusion, caffeine (10 mM) does not affect maximal isometric, fast, and slow concentric forces, stiffness and kTR in skinned fiber preparations from rabbit soleus. These results indicate that the ergogenic effect of caffeine on increasing muscle force is caused by facilitating Ca^2+^ release and not by modulating cross-bridge dynamics.
